# Several ways one goal—methanogenesis from unconventional substrates

**DOI:** 10.1007/s00253-020-10724-7

**Published:** 2020-06-15

**Authors:** Julia M. Kurth, Huub J. M. Op den Camp, Cornelia U. Welte

**Affiliations:** 1 Department of Microbiology, Institute for Water and Wetland Research, Radboud University, Heyendaalseweg 135, 6525 AJ Nijmegen, The Netherlands; 2 Soehngen Institute of Anaerobic Microbiology, Radboud University, Heyendaalseweg 135, 6525 AJ Nijmegen, The Netherlands

**Keywords:** Methane production, Extended substrate range, Novel pathways, Archaea

## Abstract

**Abstract:**

Methane is the second most important greenhouse gas on earth. It is produced by methanogenic archaea, which play an important role in the global carbon cycle. Three main methanogenesis pathways are known: in the hydrogenotrophic pathway H_2_ and carbon dioxide are used for methane production, whereas in the methylotrophic pathway small methylated carbon compounds like methanol and methylated amines are used. In the aceticlastic pathway, acetate is disproportionated to methane and carbon dioxide. However, next to these conventional substrates, further methanogenic substrates and pathways have been discovered. Several phylogenetically distinct methanogenic lineages (*Methanosphaera*, *Methanimicrococcus*, *Methanomassiliicoccus*, *Methanonatronarchaeum*) have evolved hydrogen-dependent methylotrophic methanogenesis without the ability to perform either hydrogenotrophic or methylotrophic methanogenesis. Genome analysis of the deep branching *Methanonatronarchaeum* revealed an interesting membrane-bound hydrogenase complex affiliated with the hardly described class 4 g of multisubunit hydrogenases possibly providing reducing equivalents for anabolism. Furthermore, methylated sulfur compounds such as methanethiol, dimethyl sulfide, and methylmercaptopropionate were described to be converted into adapted methylotrophic methanogenesis pathways of *Methanosarcinales* strains. Moreover, recently it has been shown that the methanogen *Methermicoccus shengliensis* can use methoxylated aromatic compounds in methanogenesis. Also, tertiary amines like choline (*N*,*N*,*N*-trimethylethanolamine) or betaine (*N*,*N*,*N*-trimethylglycine) have been described as substrates for methane production in *Methanococcoides* and *Methanolobus* strains. This review article will provide in-depth information on genome-guided metabolic reconstructions, physiology, and biochemistry of these unusual methanogenesis pathways.

**Key points:**

• *Newly discovered methanogenic substrates and pathways are reviewed for the first time.*

*• The review provides an in-depth analysis of unusual methanogenesis pathways.*

*• The hydrogenase complex of the deep branching Methanonatronarchaeum is analyzed.*

## Introduction

Methane is an important greenhouse gas with an atmospheric budget of about 600 Tg per year (Conrad 2009). About 70% of the emitted methane is produced by methanogenic archaea (Conrad 2009) underlining the importance of methanogenesis for the global carbon cycle. In addition to their role in the environment and the global carbon cycle, methanogens can be used for several applications. They gain increasing importance for energy supply and the production of high-value compounds in the chemical industry (Enzmann et al. 2018). Besides their pivotal role in biogas plants, methanogens are also used in microbial electrosynthesis using CO_2_ and electrical power to generate methane. Three main methanogenesis pathways (hydrogenotrophic, aceticlastic, and methylotrophic) have been described that share the core pathway of methanogenesis yet also differ in many aspects of their biochemistry and physiology.

**Fig. 1:**
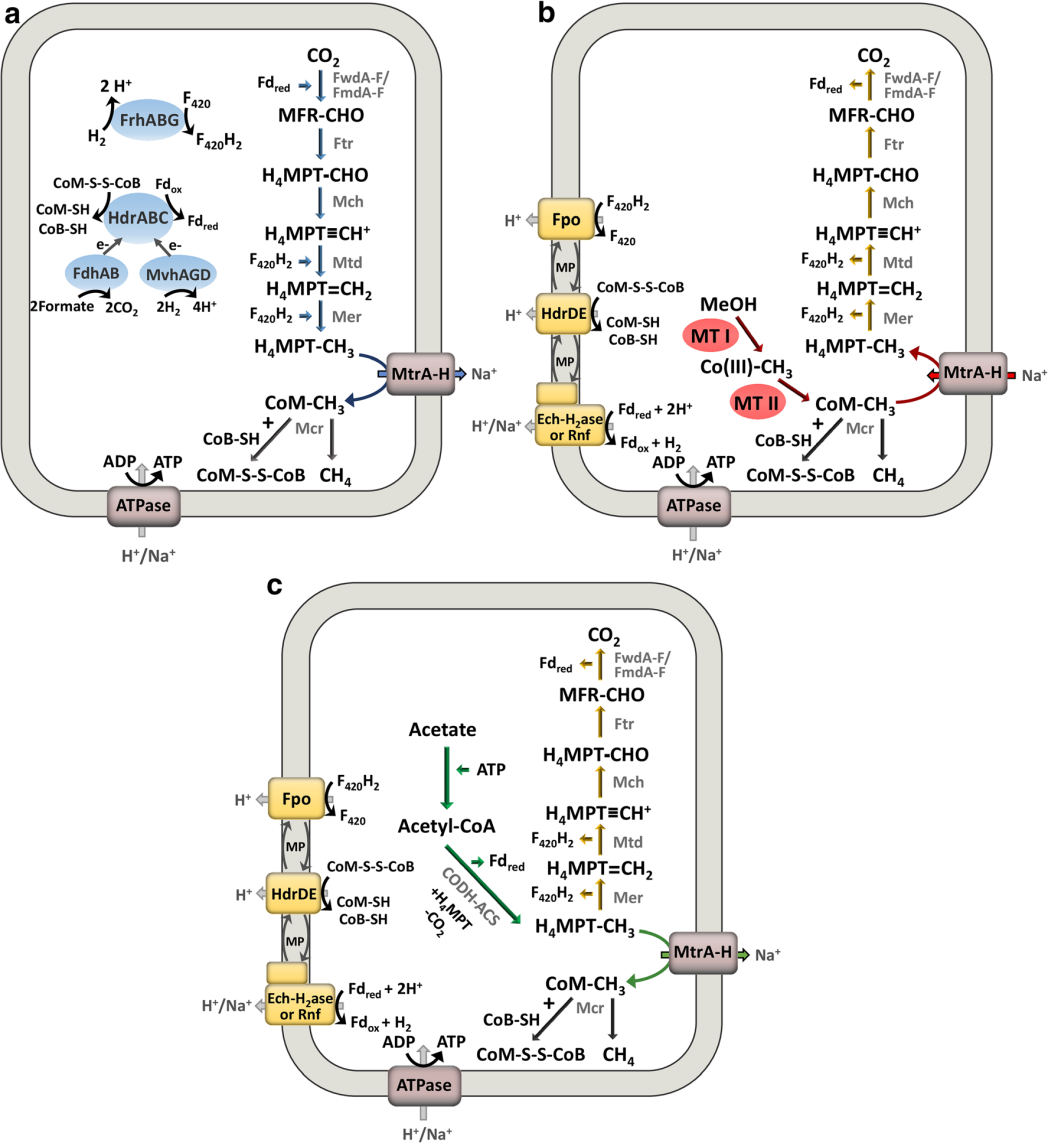
Hydrogenotrophic (**a**), methylotrophic (**b**) and aceticlastic (**c**) methanogenesis pathways. The ferredoxin electron carrier is a two-electron carrier. Some methanogens use a H_4_MPT derivative called tetrahydrosarcinopterin (H_4_SPT). The Na^+^/H^+^ translocation stoichiometry is not represented in the figure. FwdA-F/FmdA-F: formylmethanofuran dehydrogenase, Ftr: formylmethanofuran-tetrahydromethanopterin formyl-transferase, Mch: methenyl-tetrahydromethanopterin cyclohydrolase, Mtd: methylenetetrahydromethanopterin dehydrogenase, Mer: 5,10-methylenetetrahydromethanopterin reductase, MtrA-H: tetrahydromethanopterin S-methyl-transferase, McrABCDG methyl-coenzyme M reductase, FrhABG: coenzyme F_420_-reducing hydrogenase, HdrABC: soluble heterodisulfide reductase, MvhAGD: F_420_-non-reducing hydrogenase, FdhAB: formate dehydrogenase, FpoA-O: F_420_H_2_ dehydrogenase, HdrDE: membrane-bound heterodisulfide reductase, Ech-H_2_ase: energy-converting hydrogenase, Rnf: Na^+^-translocating ferredoxin:NAD^+^ oxidoreductase complex, ATPase: ATP synthase, CODH-ACS: Acetyl-CoA decarbonylase/synthase, MTI and MTII: methyltransferase, CoB: coenzyme B, CoM: coenzyme M, H_4_MPT: tetrahydromethanopterin, MFR: methanofuran, Fd: ferredoxin, F_420_H_2_: reduced coenzyme F_420_, MP: methanophenazine, CO(III): cobalamin binding protein

The largest phylogenetic diversity is found within the hydrogenotrophic methanogens. They utilize hydrogen as an electron donor for the reduction of carbon dioxide to methane (Fig. 1A). Two main hydrogenases are used for the oxidation of dihydrogen: the soluble F_420_-reducing hydrogenase Frh reduces the methanogenic cofactor F_420_ to F_420_H_2_ which is subsequently re-oxidized during the reduction of carbon dioxide to methane. The soluble Mvh hydrogenase forms a complex with a heterodisulfide reductase (HdrABC) and couples the oxidation of dihydrogen to the reduction of ferredoxin and the heterodisulfide CoM-S-S-CoB in a process called flavin-based electron bifurcation (Kaster et al. 2011). The reduced ferredoxin is required for the first step of methanogenesis, the reduction of carbon dioxide to a cofactor-bound formyl group. The CoM functions as a methyl carrier and forms the heterodisulfide together with CoB in the last step of methanogenesis. To replenish the cell with reduced ferredoxin which is also required for the biosynthesis of cell components from CO_2_ some methanogens use energy-converting hydrogenases such as Eha catalyzing the sodium motive force‑driven reduction of ferredoxin with H_2_ (Thauer 2012). Energy conservation during hydrogenotrophic methanogenesis happens exclusively during a methyl transfer reaction that is part of the core pathway of methanogenesis. The responsible membrane-bound methyltransferase Mtr translocates sodium ions across the membrane leading to the buildup of a sodium motive force that is subsequently used by an ATP synthase.

Aceticlastic methanogenesis (Fig. 1C) is performed by *Methanosarcinaceae* and *Methanotrichaceae* (formerly named *Methanosaetaceae*). Acetate enters the cell via an acetate transporter (Welte et al. 2014) and is subsequently activated to acetyl-CoA, either by the concerted action of acetate kinase and transacetylase (in *Methanosarcinaceae*) or by the activity of acetyl-CoA synthetase (in *Methanotrichaceae*, Berger et al. 2012). After acetate activation to acetyl-CoA, the molecule is dismutated through the enzyme acetyl-CoA decarbonylase/synthase: the carbonyl group is oxidized to carbon dioxide whereas the methyl group is funneled into the central methanogenic pathway in order to be reduced to methane. Energy conservation happens at the membrane-bound methyltransferase Mtr as well as in a membrane-bound electron transport chain that utilizes reduced ferredoxin and the heterodisulfide which both are produced during methanogenesis (Welte and Deppenmeier 2014). During aceticlastic methanogenesis, more Na^+^/H^+^ ions translocate during a single round of methanogenesis compared with hydrogenotrophic methanogenesis, yet the former pathway of methanogenesis also requires an initial ATP investment during the activation of acetate to acetyl-CoA.

Methylotrophic methanogenesis (Fig. 1B) is performed by members of the *Methanosarcinales*. They possess substrate-specific methyltransferase systems for the utilization of methanol and methylated amines. These enzyme systems consist of three components: a substrate-specific methyltransferase that transfers the methyl group to a corrinoid protein. Subsequently, a second methyltransferase funnels the methyl group into the methanogenic pathway at the stage of methyl-CoM. Three quarters of the methyl groups are reduced to methane whereas one quarter is oxidized to carbon dioxide, in order to generate reducing equivalents as electron donors for a membrane-bound electron transport chain that uses the heterodisulfide as an electron acceptor. Energy conservation only happens during membrane-bound electron transport, as the membrane-bound methyltransferase operates in the reverse reaction, thereby dissipating the proton/sodium motive force.

In addition, a broad range of substrates can be converted by syntrophic interaction of methanogens and bacteria that closely cooperate in methanogenic degradation. The methanogenic degradation of fatty acids, alcohols, most aromatic compounds and amino acids, and others is performed in syntrophy between fermenting bacteria and methanogenic archaea (Worm et al. 2010).

Besides the canonical methanogenesis pathways described above and syntrophic interactions of methanogens and bacteria, methanogens are capable of methane generation from additional substrates, which is much less widely known and also less appreciated in environmental studies. In this review article, we will describe the current knowledge of methanogenesis pathways that go beyond the three pathways outlined above, and identify current knowledge gaps.

## Hydrogenotrophic methanogenesis with additional electron donors

### Formate

**Fig. 2:**
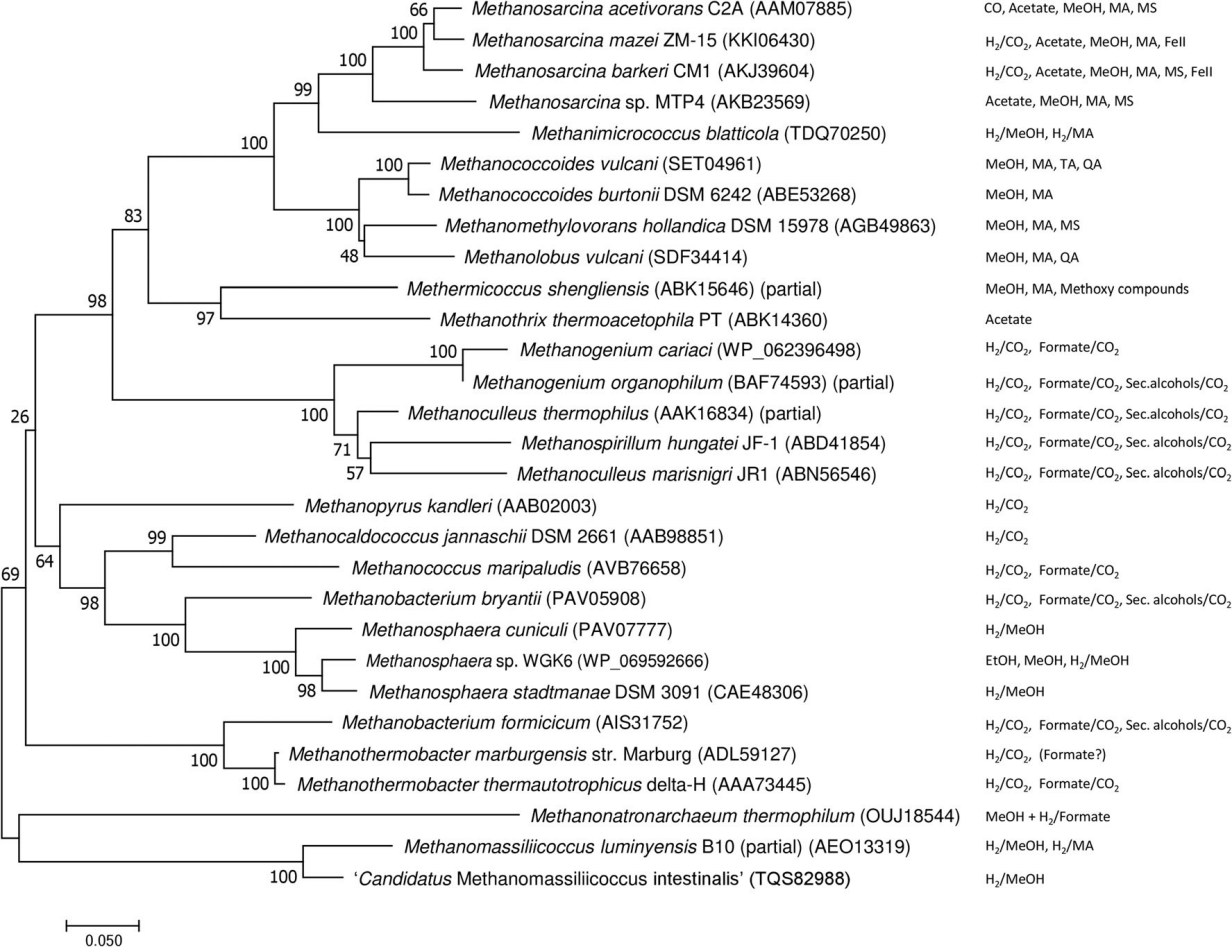
Evolutionary relationships of methyl-coenzyme M reductase (subunit A) of different methanogens. The evolutionary history was inferred using the Neighbor-Joining method. The optimal tree with the sum of branch length = 3.29201331 is shown. The percentage of replicate trees in which the associated taxa clustered together in the bootstrap test (500 replicates) are shown next to the branches. The tree is drawn to scale, with branch lengths in the same units as those of the evolutionary distances used to infer the phylogenetic tree. The evolutionary distances were computed using the Dayhoff matrix-based method and are in the units of the number of amino acid substitutions per site. The analysis involved 29 amino acid sequences. All ambiguous positions were removed for each sequence pair. There were a total of 583 positions in the final dataset. Evolutionary analyses were conducted in MEGA7 (Kumar et al. 2016). MA: methylamines, MS: methylated sulfur compounds, TA: tertiary amines, QA: quaternary amines

Many hydrogenotrophic methanogens can use formate instead of H_2_ to make methane from CO_2_ (Fig. 2). For the conversion of formate, these methanogens use the enzyme formate dehydrogenase. The activity of this enzyme, consisting of the two subunits FdhA and FdhB, leads to the production of reduced coenzyme F_420_ (Jones and Stadtman 1979; Schauer and Ferry 1982, 1986). Moreover, it has been shown that an electron bifurcating enzyme complex can couple formate oxidation to heterodisulfide reduction (Costa et al. 2010).

### Carbon monoxide

Some methanogens like *Methanosarcina acetivorans* can use carbon monoxide as a growth substrate, producing methane via a pathway that involves hydrogen as an intermediate (Rother and Metcalf 2004). *Methanosarcina acetivorans* C2A was shown to rather produce acetate and formate from CO than methane. Resting cell experiments demonstrated that methane production decreased linearly with increasing CO partial pressures, consistent with inhibition of methanogenesis by CO. Phosphotransacetylase and acetate kinase were required for growth on acetate and CO as growth substrates, i.e., aceticlastic methanogenesis and carboxydotrophic acetogenesis. Moreover, it has been found that a monofunctional carbon monoxide dehydrogenase (CODH) system contributes to, but is not required for, carboxydotrophic growth of *M. acetivorans* and that the bifunctional acetyl-CoA decarbonylase/synthase system is synthesized at elevated levels in response to CO (Rother et al. 2007). Further, CmtA (MA4384) is a soluble CH_3_-tetrahydrosarcinapterin:HS-CoM methyltransferase postulated to supplement the membrane-bound CH_3_-tetrahydrosarcinapterin:HS-CoM methyltransferase during CO-dependent growth of *M. acetivorans* (Vepachedu and Ferry 2012). Tetrahydrosarcinopterin is an analog to the C_1_-carrier tetrahydromethanopterin (H_4_MPT) which is most commonly used for C_1_-group transfer in methanogens. The soluble CmtA and homologs potentially provide a mechanism for bypassing MtrA-H, allowing growth at otherwise prohibitively low CO concentrations and equipping the cell to accommodate fluctuations in the CO concentrations that are encountered in the environment, thereby maximizing the thermodynamic efficiency for optimal ATP synthesis and growth by partitioning methyl transfer through CmtA and MtrA-H (Vepachedu and Ferry 2012).

### Ethanol

Some *Methanobrevibacter* sp. (*Methanobacteriales*) are capable of using ethanol as an electron donor for CO_2_ reduction to methane, yet not in the absence of hydrogen (Leahy et al. 2013; Poehlein et al. 2018). They possess the *walC* and *walD* alcohol and aldehyde dehydrogenase genes which enable them to convert ethanol to acetate. Also, a member of the *Methanomicrobiales*, *Methanofollis ethanolicus*, is able to grow with CO_2_ and ethanol as a electron donor (Imachi et al. 2009). The *M. ethanolicus* genome (Narihiro et al. 2016) encodes three sets of alcohol and aldehyde dehydrogenases (iron-dependent alcohol dehydrogenases: MEFOE_RS00535, MEFOE_RS00570, MEFOE_RS02725; aldehyde dehydrogenases: MEFOE_RS06760, MEFOE_RS07165, MEFOE_RS03840). Several *Methanosarcina* and *Methanoculleus* genomes encode homologs to the *M. ethanolicus* alcohol and aldehyde deydrogenases indicating that the trait of using ethanol as electron donor might be more widespread; however, these genome-based predictions need to be experimentally validated. A *Methanosphaera* strain isolated from the kangaroo foregut was capable of reducing methanol with ethanol as a electron donor (Hoedt et al. 2016); as this methanogen is not capable of hydrogenotrophic methanogenesis, it will be discussed under “hydrogen-dependent methylotrophic methanogenesis.”

### Propanol/2-butanol

**Fig. 3:**
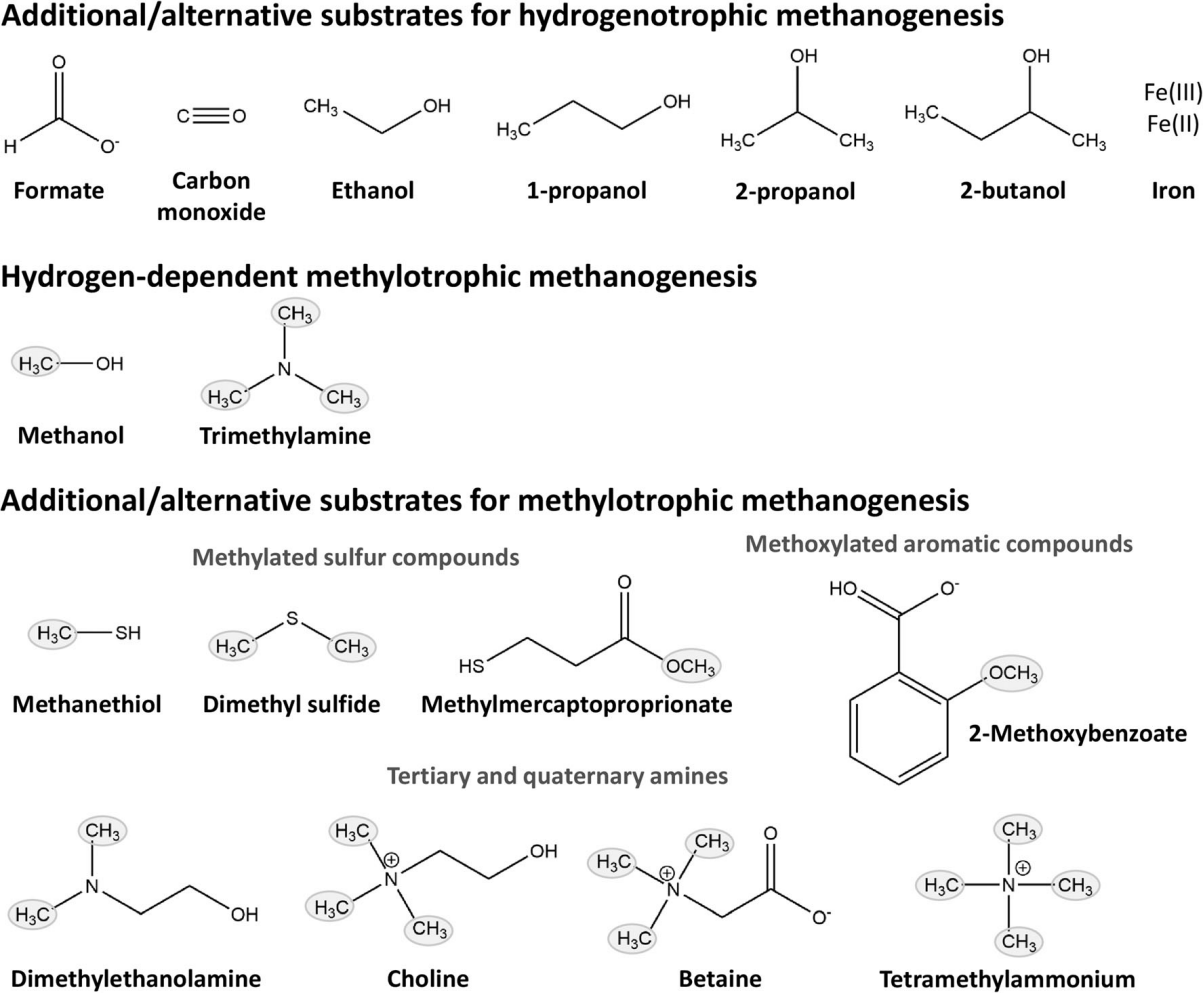
Extended substrate range of methanogens. 2-Methoxybenzoate is only one example for methoxylated aromatic compounds that can be used for methanogenesis (Mayumi et al. 2016)

Three methanogen strains, two mesophilic, and one thermophilic strain, were isolated with 2-propanol as the hydrogen donor for methanogenesis from CO_2_ (Widdel 1986; Widdel et al. 1988). The strains were designated *Methanogenium thermophilum*, *Methanogenium organophilum*, and *Methanospirillum hungatei*. One mole of CH_4_ was formed by CO_2_ reduction, with four moles of 2-propanol being converted to acetone. In addition to 2-propanol, the isolates used 2-butanol, H_2_, or formate and one strain even ethanol and 1-propanol (Fig. 3; Widdel 1986); however, growth was poor compared to the use of H_2_ and CO_2_ as substrates (Zellner and Winter 1987). Each secondary alcohol was oxidized to its ketone. Other methanogens like *Methanobacterium formicicum*, *Methanogenium marisnigri*, *Methanospirillum hungatei* strain GP1, *Methanobacterium bryantii*, *Methanomicrobium paynteri*, and *Methanocorpusculum parvum* were also able to grow on secondary alcohols (Zellner and Winter 1987; Widdel et al. 1988). The genome of *Methanospirillum hungatei* JF-1 (Gunsalus et al. 2016) contains one gene for an iron-dependent alcohol dehydrogenase (MHUN_RS00885) that is also present in other *Methanospirillum* genomes and furthermore similar to alcohol dehydrogenases found in sulfate-reducing bacteria. The genome of *Methanogenium cariaci* JCM10550 also encodes for an iron-containing alcohol dehydrogenase (JCM10550_RS03520) which is about 50% identical to the *Methanospirillum* protein. Homologs of these alcohol dehydrogenases are found in other members of the *Methanomicrobiales* (*Methanoplanus*, *Methanolacinia*, *Methanoculleus*, *Methanofollis*). Whether this enzyme is responsible for the conversion of secondary alcohols needs to be determined experimentally.

### Iron

Aceticlastic methanogenesis by *Methanosarcina mazei* is accelerated by magnetite and is correlated with the redox cycling of Fe(II) and Fe(III) in the mineral (Wang et al. 2020). The genomic analysis predicts that in addition to electron transfer components essential for aceticlastic methanogenesis, *Ms. mazei* contains an outer surface multiheme *c*-type cytochrome (MHC) and a few function-unknown surface proteins that harbor monoheme motifs. It is hypothesized that the redox cycling of nanoFe_3_O_4_ delivers a positive influence via the MHC to the membrane electron transfer chain and hence promotes aceticlastic methanogenesis.

Two species (*M. barkeri* and *Methanococcus voltae*) reduced significant amounts of Fe(III) oxide using hydrogen as the electron donor, and 0.1 mM of anthraquinone-2,6-disulphonate (AQDS) as soluble electron shuttle in the medium greatly accelerated Fe(III) reduction by these organisms (Bond and Lovley 2002). Moreover, it was demonstrated that electrons were transferred to Fe(III) by hydrogen-utilizing methanogens even when growth and methanogenesis were inhibited.

### Hydrogen-dependent methylotrophic methanogenesis

Next to the three common methanogenesis pathways, some methanogens use a mixed-mode of methanogenesis by combining the hydrogenotrophic and the methylotrophic pathway, which is seen as a novel mode of energy metabolism in methanogenic archaea. Such an H_2_-dependent methylotrophic methanogenesis pathway is used in *Methanosphaera stadtmanae*, belonging to the *Methanobacteriales*, *Methanimicrococcus blatticola*, belonging to the *Methanosarcinales*, in *Methanomassiliicoccales* like *Methanomassilicoccus luminyensis*, or in *Methanonatronarchaeales* like *Methanonatronarchaeum thermophilum.* These methanogens have in common that they lack (or do not express) the upper part of the methanogenesis pathway in which cofactor-bound methyl groups are oxidized to carbon dioxide or vice versa, explaining why they are incapable of methanogenesis from either methylated compounds or H_2_ + CO_2_ alone. In addition, all of those organisms—except the deep branching *Methanonatronarchaeum*—have in common that they thrive in a gut system and therefore this type of metabolism might be an adaption to this specific environment.

**Fig. 4:**
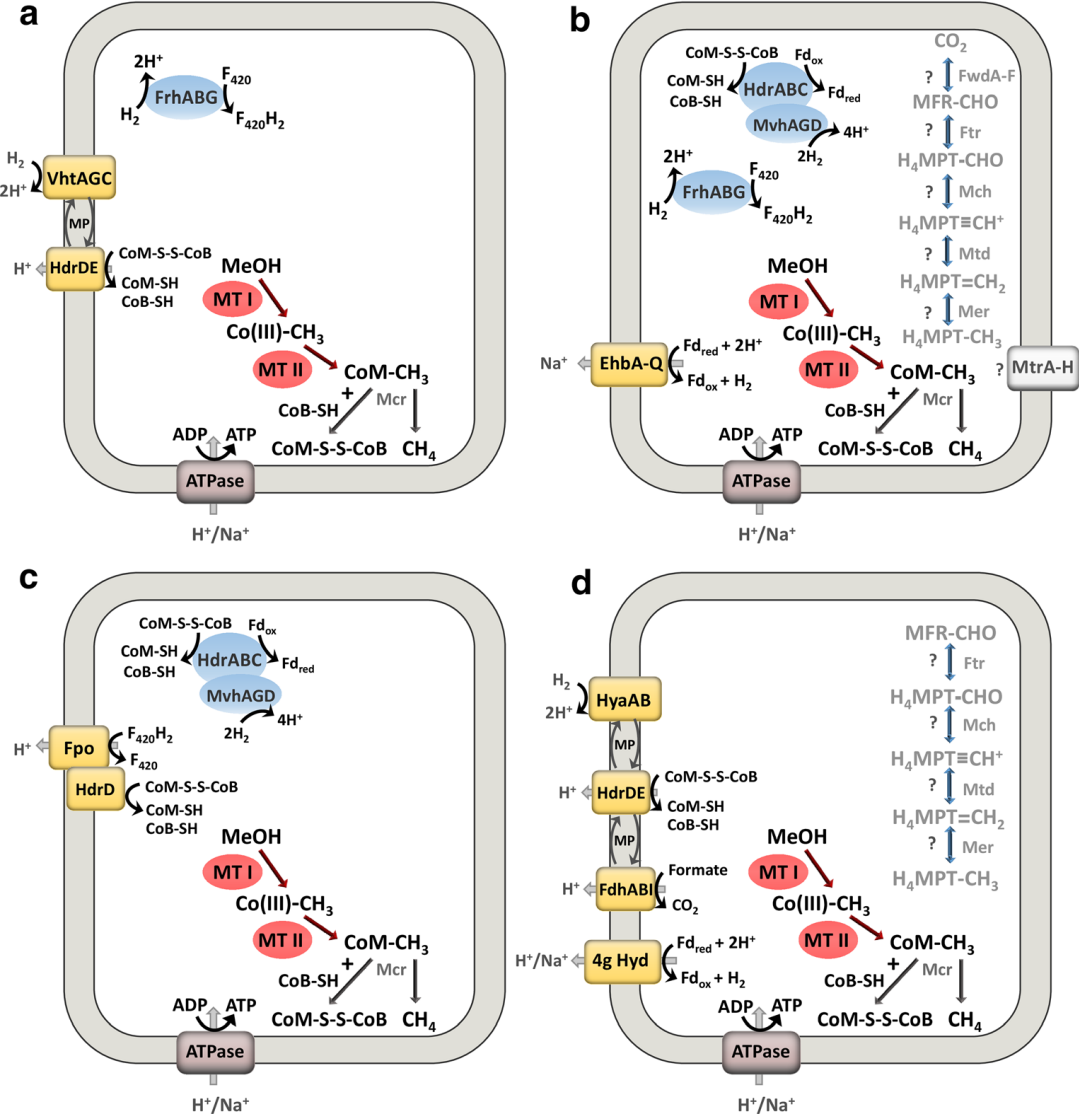
Methanogenesis pathway in *Methanimicrococcus blatticola* (**a**), *Methanosphaera stadtmanae* (**b**), *Methanomassiliicoccus luminyensis* (**c**), and *Methanonatronarchaeum thermophilum* (**d**) Question marks mark proteins which are encoded in the distinctive genome, but their abundance and function in the cell are yet unclear. The ferredoxin electron carrier is a 2-electron carrier. Some methanogens use a H_4_MPT derivative called tetrahydrosarcinopterin (H_4_SPT). The Na^+^/H^+^ translocation stoichiometry is not represented in the figure. FwdA-F/FmdA-F: formylmethanofuran dehydrogenase, Ftr: formylmethanofuran-tetrahydromethanopterin formyl-transferase, Mch: methenyl-tetrahydromethanopterin cyclohydrolase, Mtd: methylenetetrahydromethanopterin dehydrogenase, Mer: 5,10-methylenetetrahydromethanopterin reductase, MtrA-H: tetrahydromethanopterin S-methyl-transferase, McrABCDG methyl-coenzyme M reductase, FrhABG: coenzyme F_420_-reducing hydrogenase, HdrABC: soluble heterodisulfide reductase, MvhAGD: F_420_-non-reducing hydrogenase, FdhABI: formate dehydrogenase (FdhI contains a b-type heme), FpoA-O: F_420_H_2_ dehydrogenase, HdrDE: membrane bound heterodisulfide reductase, EhbA-Q: energy-conserving hydrogenase, VhtGACD: [NiFe]-hydrogenase, HyaAB: H_2_-producing hydrogenase, 4 g Hyd: 4 g-type hydrogenase, ATPase: ATP synthase, MTI and MTII: methyltransferase, CoB: coenzyme B, CoM: coenzyme M, H_4_MPT: tetrahydromethanopterin, MFR: methanofuran, Fd: ferredoxin, F_420_H_2_: reduced coenzyme F_420_, MP: methanophenazine, CO(III): cobalamin binding protein.

*Methanimicrococcus blatticola* was isolated from the hindgut of a cockroach and produces methane by the reduction of methanol and methylated amines with molecular hydrogen (Sprenger et al. 2000). It was shown that *M. blatticola* lacks the pathway for methyl-CoM oxidation to CO_2_ (Fig. 4A), explaining the requirement of hydrogen for methane production from methanol or methylated amines and the obligate heterotrophy of the organism (Sprenger et al. 2005). A further observation was that the reduction of CoM-S-S-CoB was associated with the membrane fraction of this organism hinting towards the presence of the membrane-associated heterodisulfide reductase subunit HdrD. Moreover, a hydrogen-dependent reduction of CoB-S-S-CoM could mainly be associated with the membrane fraction (Sprenger et al. 2005). Recently, the full genome sequence of *M. blatticola* became available, providing novel insights into the energy conservation during H_2_-dependent methylotrophic methanogenesis in this organism. The genome encodes a membrane-bound methanophenazine-reducing hydrogenase homologous to the cytochrome *b* containing NiFe-hydrogenase (Vht hydrogenase) found in *Methanosarcina* (encoded by the gene cluster C7391_RS00170-C7391_RS00185) as well as a cytoplasmic F_420_-reducing hydrogenase (C7391_RS03665–3670) (Fig. 4A, Table 1). In addition, a membrane-bound heterodisulfide reductase consisting of the subunits HdrDE was detected (C7391_RS01355, C7391_RS01360). No genes encoding for an F_420_H_2_ dehydrogenase (Fpo complex) or an Ech/Eha/Ehb hydrogenase could be identified which often plays a role in regenerating F_420_ and oxidized/reduced ferredoxin. These results indicate that energy conservation is happening at a simple H_2_-dependent respiratory chain where H_2_ is oxidized by the methanophenazine-reducing hydrogenase, followed by electron shuttling through methanophenazine to the membrane-bound heterodisulfide reductase. Both enzyme complexes contribute to the formation of a proton gradient that can subsequently be used for ATP synthesis.

**Table 1:** Key enzymes for methanogenesis from unusual substrates

Organism	Protein	Gene identifier	Protein identifier
*Methanomassilicoccus luminyensis* H_2_ + MeOH	HdrD	WYS_RS12230	WP_019178460
	FpoNMLKJJIHDCBA	WYS_RS00365-420	WP_019176172-83
	HdrABC MvhAGD	WYS_RS07030, WYS_RS08335, WYS_RS08340, WYS_RS07015, WYS_RS07020, WYS_RS07025	WP_026068905, WP_019177711, WP_026068956, WP_019177457, WP_026068904, WP_019177459
*Methanosphaera stadtmanae* H_2_ + MeOH	EhbA-P, EhbQ	Msp_1457-42, Msp_1436	ABC57827-12, ABC57807
	HdrA1B1C1	Msp_1476, Msp_1013, Msp_1014	ABC57846, ABC57401, ABC57402
	HdrA2B2C2	Msp_0127-25	ABC56545-43
	MvhDGA	Msp_0314-16	ABC56724-26
	FrhADGB	Msp_1302-05	ABC57679-82
*Methanimicrococcus blatticola* H_2_ + MeOH	VhtGACD FrhADGB HdrDE	C7391_RS00170-85, C7391_RS03655-70, C7391_RS01355-60	WP_133516533-36, WP_133517202-05, WP_133516741-42
*Methanonatronarchaeum thermophilum* H_2_ + MeOH	HdrDE HyaABD FdhABDI Multisubunit hydrogenase	AMET1_RS05590–95, AMET1_RS03000–05, AMET1_RS02990, AMET1_RS00565–70, AMET1_RS01775, AMET1_RS02995, AMET1_RS07305-75	WP_086637509-10, WP_086637000-01, WP_086636998, WP_086636646, WP_086636543, WP_161490715, WP_086636999, WP_143406895, WP_086637820, WP_086637822, WP_161490830, WP_086637824-25, WP_086637860, WP_086637826-32
*Methanosarcina acetivorans* Methyl sulfides	MtsD	MA_0859	AAM04298
	MtsF	MA_4384	AAM07726
	MtsH	MA_4558	AAM07897
Methylmercaptopropionate	MtpCAP	MA_4164–66	AAM07512–14
*Methanomethylovorans hollandica* Methyl sulfides	MtsD, MtsF, MtsH, MtpCAP	METHO_RS06035 METHO_RS01750 METHO_RS03770 METHO_RS05605	WP_015324657 WP_015323796 WP_015324192 WP_015324567
*Methanosarcina* MTP4 Methyl sulfides	MtsD MtsF MtsH MtpCAP	MSMTP_RS14200 MSMTP_RS14235 MSMTP_RS00505 MSMTP_RS03110-20	WP_048180685 WP_048180700 WP_048177073 WP_052718253 WP_048177774 WP_082090704
*Methanosarcina semesiae* Methyl sulfides	?	?	?
*Methermicoccus shengliensis* ZC-1 Methoxylated aromatic compounds	MtvB O-demethylase	BP07_RS03255	WP_042685518
	MtvB O-demethylase	BP07_RS03250	WP_042685515
	Corrinoid protein	BP07_RS03260	WP_042685521
	MtrH-like methyltransferase	BP07_RS03240	WP_042685937
	Corrinoid activation protein	BP07_RS03235	WP_042685513
*Methanococcoides* Tertiary amines	?	?	?
*Methanolobus vulcani* Quaternary amines	MtgB methyltransferase	FKV42_RS08545	WP_154809802
	Corrinoid protein	FKV42_RS08550	WP_154809803
	Corrinoid activator	FKV42_RS10455	WP_154810143
	CoM methyltransferase	FKV42_RS10480	WP_154810148

**Fig. 5:**
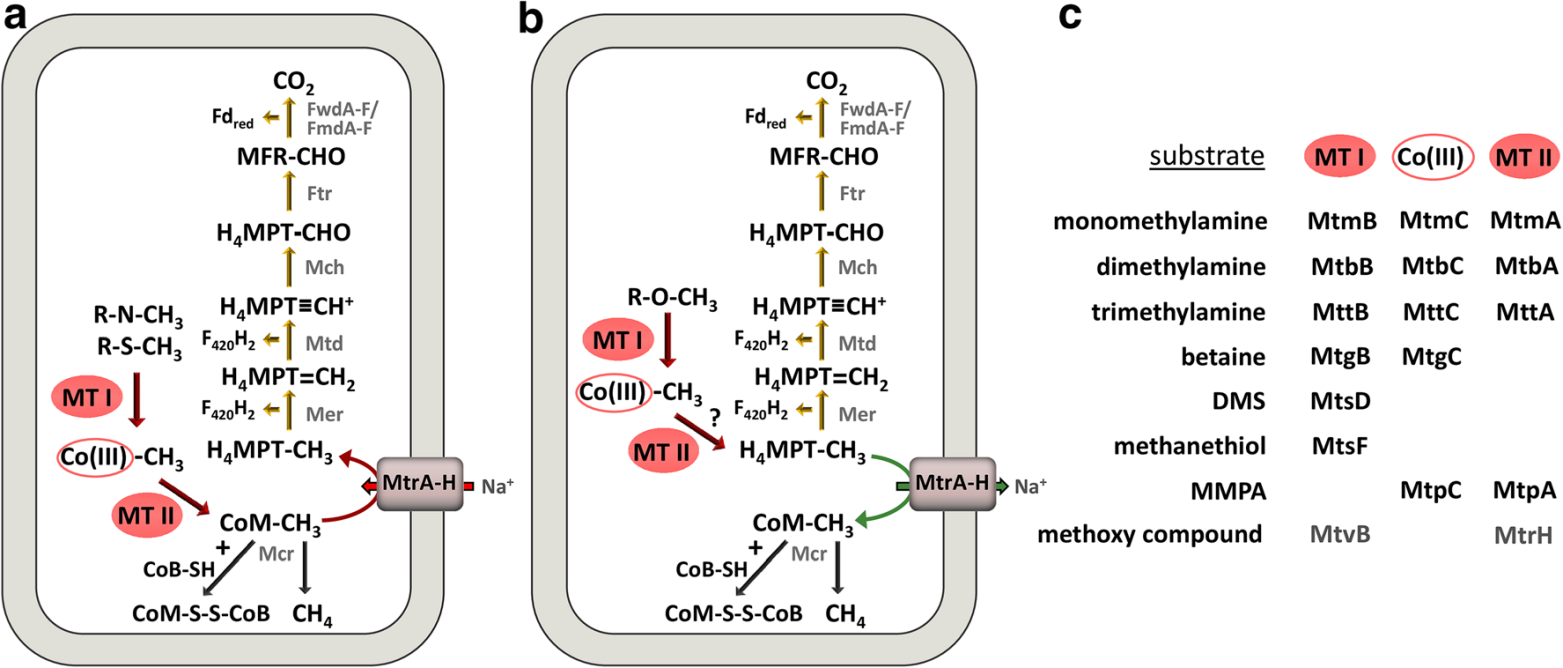
Methanogenesis from methylated sulfur compounds or tertiary and quaternary amines (**a**) and from methoxylated aromatic compounds in *Methermicoccus shengliensis* (**b**). Panel **c** shows the proteins that are involved in methyl transfer for diverse substrates. The question mark indicates that there is no biochemical evidence yet if the methyl group is transferred to H_4_MPT or CoM during growth on methoxy compounds. For growth on methoxy compounds proteins similar to the O-demethylase MtvB and the methyltransferase MtrH are most likely involved in the methyl transfer. Some methanogens use a H_4_MPT derivative called tetrahydrosarcinopterin (H_4_SPT). The Na^+^/H^+^ translocation stoichiometry is not represented in the figure. FwdA-F/FmdA-F: formylmethanofuran dehydrogenase, Ftr: formylmethanofuran-tetrahydromethanopterin formyl-transferase, Mch: methenyl-tetrahydromethanopterin cyclohydrolase, Mtd: methylenetetrahydromethanopterin dehydrogenase, Mer: 5,10-methylenetetrahydromethanopterin reductase, MtrA-H: tetrahydromethanopterin S-methyl-transferase, McrABCDG methyl-coenzyme M reductase, MTI, and MTII: methyltransferase, CoB: coenzyme B, CoM: coenzyme M, H_4_MPT: tetrahydromethanopterin, MFR: methanofuran, CO(III): cobalamin binding protein, MtrH: tetrahydromethanopterin S-methyltransferase subunit H

For the organisms conducting hydrogen-dependent methylotrophic the enzymes important for energy conversion/recycling of reducing equivalents are shown as those play an important role of the special metabolism of those organisms and they are also depicted in Fig. 4. For the other organisms, we focused on the enzymes mentioned in the text and/or shown in Fig. 5. EhbA-Q: energy-conserving hydrogenase, VhtGACD: [NiFe]-hydrogenase, FpoA-O: F_420_H_2_ dehydrogenase, HdrDE: membrane-bound heterodisulfide reductase, HdrABC: soluble heterodisulfide reductase, MvhAGD: F_420_-non-reducing hydrogenase, FrhADGB: coenzyme F_420_-reducing hydrogenase, MtrH: tetrahydromethanopterin S-methyltransferase subunit, MtsDFH: proteins involved in the catabolism of dimethylsulfide, MtpCAP: proteins involved in the catabolism of methylmercaptopropionate (MMPA). MtvB: O-demethylase, MtgB methyltransferase

*Methanosphaera stadtmanae* has been isolated from the human gut and also strictly requires hydrogen for methane production from methanol (Miller and Gennis 1985). Although the organism still encodes for most of the genes required for methyl oxidation to CO_2_, it has lost the capability to oxidize methanol to CO_2_ and vice versa (Fig. 4B; van de Wijngaard et al. 1991; Fricke et al. 2006). The remaining proteins of the upper methanogenesis pathway are assumed to be used in other metabolic pathways like purine and amino acid metabolism (van de Wijngaard et al. 1991). Acetyl-CoA decarbonylase/synthase is absent in *M. stadtmanae* which is why it cannot synthesize acetate from inorganic precursors, explaining its requirement for acetate in the growth medium (Fricke et al. 2006). In the genome of *M. stadtmanae* genes for three different hydrogenases are found: the F_420_-reducing hydrogenase FrhADGB (Msp_1302 to Msp_1305), the cytoplasmic F_420_-nonreducing hydrogenase MvhDGA (Msp_0314 to Msp_0316) and the energy-conserving hydrogenases EhbABCDEFGHIJKLM-NOPQ (Msp_1457 to Msp_1442 and Msp_1436) (Fricke et al. 2006). Moreover, two soluble HdrABC complexes are encoded in the genome (HdrA1B1C1: Msp_1476 and Msp_1013/4; HdrA2B2C2: Msp_0125-7). It is assumed that a soluble hydrogenase/heterodisulfide reductase complex (MvhADG/HdrABC) is used in the organism to transfer the electrons resulting from H_2_ oxidation by MvhADG to the reduction of heterodisulfide and ferredoxin by HdrABC (Fricke et al. 2006; Thauer et al. 2008). The reduced ferredoxin then could be reoxidized by the membrane-bound Ehb complex resulting in a sodium motive force (Thauer et al. 2008). The membrane-bound Mtr complex does not contribute to energy conservation in *M. stadtmanae*. Interestingly, a different phylotype of *Methanosphaera* isolated from the kangaroo foregut was capable of reducing methanol with ethanol as the electron donor, in the absence of hydrogen (Hoedt et al. 2016). Methanogenesis from ethanol alone was not observed. The genome of *Methanosphaera* sp. WGK6 encodes alcohol and aldehyde dehydrogenases that provide electrons for the reduction of methanol to methane. How this metabolism is coupled with energy conservation remains to be elucidated.

*M. luminyensis* was isolated from human feces and was found to only produce methane when both hydrogen and methanol were present (Dridi et al. 2012). It is phylogenetically distant from other methanogens and affiliated with the *Thermoplasmatales*. Next to methanol *Methanomassiliicoccales* can also reduce methylamines in the presence of hydrogen (Lang et al. 2015). In contrast to *M. stadtmanae*, the *M. luminyensis* genome lacks the entire pathway for CO_2_ reduction to methyl coenzyme M (Gorlas et al. 2012). In contrast to hydrogenotrophic methanogens *M. luminyensis* does not possess the energy-conserving methyltransferase (MtrA-H) to generate a sodium motive force and contrary to methylotrophic methanogens it does not produce cytochromes for energy conservation. Instead, *Methanomassiliicoccales* possess a F_420_:methanophenazine oxidoreductase (Fpo) which lacks the F_420_-oxidizing subunit FpoF comparable to the ferredoxin-dependent Fpo-like homolog in *Methanothrix thermoacetophila* (Welte and Deppenmeier 2011). Moreover, *Methanomassiliicoccales* also lack the subunit HdrE of the membrane-bound heterodisulfide reductase HdrDE (Fig. 4C, Table 1). Therefore, it is assumed that in those organisms the Fpo-like complex interacts directly with subunit HdrD, forming an energy-converting ferredoxin:heterodisulfide oxidoreductase (Lang et al. 2015). Both heterodisulfide reductases HdrABC and HdrD as well as the “headless” ferredoxin-dependent F_420_:methanophenazine oxidoreductase Fpo are highly transcribed in *M. luminyensis* (Kröninger et al. 2016). In addition, the activity of the HdrABC/MvhADG complex and of HdrD was measured. It is proposed that the membrane-bound electron transfer is based on the conversion of two molecules of methanol resulting in the formation of two molecules of the heterodisulfide (Kröninger et al. 2016). The HdrABC/MvhADG complex catalyzes the H_2_-dependent reduction of heterodisulfide and the formation of reduced ferredoxin. The reduced ferredoxin is then oxidized by the ‘headless’ Fpo complex thereby translocating up to 4 H^+^ across the membrane and electrons are channeled to HdrD for reduction of the second heterodisulfide (Kröninger et al. 2019). Interestingly, protons instead of Na^+^ ions are used as coupling ions for the generation of the electrochemical ion gradient in *Methanomassiliicoccus*.

Also, members of the *Methanonatronarchaeales* like *Methanonatronarchaeum thermophilum* have been shown to utilize C_1_ methylated compounds as electron acceptors and H_2_ or formate as electron donors (Sorokin et al. 2017, 2018). They are extremely halophilic methanogens affiliated with a new methanogenic class, the *Methanonatronarchaeia*. Although some of the enzymes that are part of the oxidative branch of methanogenesis from the methyl group to CO_2_ are encoded in the genome of the organism (*mer*, *mtd*, *mch*, *ftr*), other enzymes essential for this pathway like Mtr and Fwd are not encoded in the genome. The question of the physiological role of those remaining genes rises as the absence of the methyltransferase Mtr disconnects the methyl reducing pathway to methane from the oxidative branch of methanogenesis to CO_2_ (Fig. 4D; Ferrer et al. 2018). Sorokin et al. (2017) report a metabolic model based on genome reconstruction, suggesting that energy conservation is performed via a membrane-bound respiratory chain: membrane-bound hydrogenase (or formate dehydrogenase) acts as electron input module, and electrons are transferred to methanophenazine. The membrane-bound heterodisulfide reductase HdrDE acts as a terminal reductase and reduces the CoM-S-S-CoB heterodisulfide. Interestingly, this respiratory chain contains cytochromes, which were previously only found in the *Methanosarcinales* within the methanogens. In the current reconstruction of the metabolic model, it is unclear how electrons are transferred from methanogenesis to anabolic reactions, as cytoplasmic hydrogenases are absent and no enzymes for reduction of F_420_, ferredoxin or NAD(P)^+^ have been detected. Intriguingly, we identified a gene cluster encoding proteins related to membrane-bound NADH dehydrogenase subunits (AMET1_RS07305 to AMET1_RS07375).. Methanogens encode multisubunit membrane-bound hydrogenases, also distantly related to NADH dehydrogenases, termed Eha, Ehb, or Ech, classified as group 4h, 4i, and 4e hydrogenases, respectively, according to Søndergaard et al. (2016). They have a role in anaplerotic reactions to provide low-potential electrons with H_2_ as an electron donor (Eha, Ehb, Major et al. 2010; Lie et al. 2012) or in ferredoxin-dependent energy conservation (Welte et al. 2010). According to HydDB (Søndergaard et al. 2016), the putative multisubunit hydrogenase of *Methanonatronarchaeum* is classified as group 4g hydrogenase for which no experimental data are available. Our analysis suggests that this class 4g membrane-bound hydrogenase is involved in providing low-potential electrons for anabolism and provides the missing link between catabolism and anabolism. This hypothesis needs to be addressed by future biochemical and physiological experiments.

Next to the previously described methanogens specialized in H_2_-dependent methylotrophic growth, there are also versatile methanogens that can additionally grow on hydrogen and methanol as, e.g., *Methanosarcina barkeri. M. barkeri* has been shown to consume hydrogen and methanol in equimolar amounts for energy conservation and is under these growth conditions dependent on acetate for anabolism (Mueller et al. 1986).

### Use of methylated sulfur compounds as substrates for methanogenesis

Methanethiol (MT) and dimethyl sulfide (DMS) are the dominant volatile organic sulfur compounds in freshwater sediments. In these habitats, DMS and MT formation mainly occur through sulfide methylation by anaerobic O-demethylation of methoxylated aromatic compounds (Bak et al. 1992; Lomans et al. 2001, 2002). The major part of the produced MT and DMS is degraded anaerobically by methanogens (Lomans et al. 1999b, c, 2002). Using methylated sulfur compounds for methanogenesis is energetically less favorable than using methanol as a substrate (Table 2). *Methanomethylovorans hollandica* strain DMS1T has been isolated from freshwater sediments on DMS and found to use methanol, methylamines, MT, and DMS as substrates (Lomans et al. 1999a). Methanogens like strain DMS1T can also be involved in the formation of DMS through methylation of MT as the DMS conversion is reversible (Lomans et al. 1999a). Another methanogen that is able to use DMS and methanethiol as substrates for methanogenesis is *Methanosarcina semesiae* MD1T which has been isolated from mangrove sediment (Lyimo et al. 2000). In the marine environment, DMS is the most important volatile sulfur compound and originates mainly from the algal osmolyte dimethylsulfoniopropionate (DMSP) (van der Maarel and Hansen 1997). Next to DMS also methylmercaptopropionate (MMPA) is a conversion product of DMSP in anoxic marine sediments. An example of a marine DMS degrader is the methanogen *Methanosarcina* sp. strain MTP4 which is able to use DMS as sole source of energy (Finster et al. 1992). Furthermore, it has been described for anoxic aquatic sediments that methanogens and sulfate reducers compete for DMS when it is present at low concentrations (Kiene et al. 1986; Lyimo et al. 2009) and that methanogens are the main DMS converters at high DMS concentrations (Kiene et al. 1986), whereby MT is a transient intermediate of DMS metabolism. The methylated sulfur compound MMPA that is present in the marine environment has been shown to be demethylated to mercaptopropionate and methane by three marine *Methanosarcina* strains (van der Maarel and Hansen 1997). The specific mechanism of methyl transfer from methylated sulfur compounds and the transfer into the methanogenesis pathways has not been studied for the methanogens mentioned above. In methylotrophic methanogens, the methyl group from methylated compounds like methanol is channeled into the methanogenesis pathway via transfer from the substrate to coenzyme M (CoM) (Fig. 5). It has been shown for *Methanosarcina barkeri*, a very versatile methanogen using hydrogenotrophic, methylotrophic, and aceticlastic methanogenesis, that this organism can convert DMS and MMPA to methane when grown on acetate (Paul and Krzycki 1996; Tallant and Krzycki 1996, 1997). For this organism, it has been demonstrated that a 30-kDa corrinoid protein (MtsB) and a 41-kDa protein (MtsA), forming a 480 kDa complex, are used for coenzyme M methylation by methylated thiols (Paul and Krzycki 1996; Tallant and Krzycki 1996, 1997). A closely related methanogen, *Methanosarcina acetivorans*, has been shown to be able to use DMS as the sole energy source and specific methyltransferases, called MtsD, MtsF, and MtsH (MA0859, MA4384, and MA4558) could be associated with the ability to grow on methyl sulfides (Oelgeschläger and Rother 2009a, b). Further analysis of those methyltransferases revealed that the preferred substrate for MtsD is DMS, while the preferred substrate for MtsF is methanethiol and MtsH appears to accept both substrates (Fu and Metcalf 2015). Interestingly, all three *M. acetivorans* proteins retrieve the *Methanomethylovorans hollandica* best BLAST hit METHO-RS06035 indicating that this protein might accept both methanethiol and DMS as substrates. Moreover, it has been shown that a four-gene locus, *mtpCAP*-*msrH*, is required for growth on MMPA in *M. acetivorans* (Fu and Metcalf 2015). *MtpC*, *mtpA*, and *mtpP* encode a putative corrinoid protein, a coenzyme M methyltransferase, and a major facilitator superfamily transporter, while *msrH* encodes a putative transcriptional regulator.

**Table 2:** Gibbs free energy values for different methanogenesis substrates

Substrate	Reaction equation	ΔG′° (kJ/mol CH_4_)
H_2_ + CO_2_	4 H_2_ + CO_2_ → CH_4_ + 2 H_2_O	− 131 (a)
HCOO^−^	4 HCOO^−^ + 4 H^+^ → CH_4_ + 3 CO_2_ + 2 H_2_O	− 145 (a)
CH_3_CH_2_OH + CO_2_	2 CH_3_CH_2_OH + CO_2_ → 2 CH_3_COOH + CH_4_	− 121 (e)
H_2_ + CH_3_OH	CH_3_OH + H_2_ → CH_4_ + H_2_O	− 113 (e)
CH_3_OH + CH_3_CH_2_OH	2 CH_3_OH + CH_3_CH_2_OH → 2 CH_4_ + H_2_O + CH_3_COOH	− 100 (b)
CH_3_CHOHCH_3_+ CO_2_	4 CH_3_CHOHCH_3_ + HCO_3_ + H^+^ → 4 CH_3_COCH_3_ + CH_4_ + 3 H_2_O	− 37 (c)
CH_3_OH	4 CH_3_OH → CO_2_ + 3 CH_4_ + 2 H_2_O	− 107 (a)
CH_3_-COOH	CH_3_COOH → CO_2_ + CH_4_	− 36 (a)
CH_3_-SH (CH_3_‑S‑R)	4 CH_3_SH + 3 H_2_O → 3 CH_4_ + HCO_3_^**−**^ + 4 HS^**−**^ + 5 H^+^	− 49 (b)
Betaine (CH_3_‑N‑R)	4 (CH_3_)_3_N^+^CH_2_COO^−^ + 2 H_2_O → 4 (CH_3_)_2_N^+^CH_2_COO^−^ + 3 CH_4_ + CO_2_	− 241 (c)
Choline (CH_3_‑N‑R)	4 (CH_3_)_3_N^+^CH_2_CH_2_OH + 6 H_2_O → 4 H_2_NCH_2_CH_2_OH + 9 CH_4_ + 3 CO_2_ + 4 H^+^	− 63 (d)
Trimethylamine (CH_3_‑N‑R)	4 (CH_3_)_3_N + 6 H_2_O + 4 H^+^ → 4 NH_4_^+^ + 9 CH_4_ + 3 CO_2_	− 31 (d)
2-methoxyphenol (CH_3_‑O‑R)	4 2-methoxyphenol + 2 H_2_O → 4 2-hydroxyphenol + CO_2_ + 3 CH_4_	− 90 (f)

The values for the standard free energy change (ΔG′°) are derived from (a) (Thauer 1998), (b) (Finster et al. 1992), (c) (Watkins et al. 2014), (d) (Watkins et al. 2012). (e) are values calculated by the webtool eQuilibrator (Flamholz et al. 2012) and (f) gives values calculated by use of the standard free energies of formation at 25 °C

### Methanogenesis from methoxylated aromatic compounds

Methoxylated compounds are derived from lignin and occur in large quantities on earth (De Leeuw and Largeau 1993). For a long time, it has been known that methoxylated aromatic compounds can be converted to methane by anaerobic microorganisms (Healy and Young 1979). Acetogenic bacteria were the first anaerobes discovered to use methoxylated aromatic compounds for energy conservation (Bache and Pfennig 1981) via conversion of the methyl group to acetate in the acetyl-CoA (Wood-Ljungdahl) pathway. One example is *Parasporobacterium paucivorans* that has been shown to produce MT, DMS, acetate, and butyrate from the methoxylated aromatic compound syringate (Lomans et al. 2001). However, it has recently been discovered that methanogens are also capable of using methoxylated aromatic compounds as substrate: the methanogen *Methermicoccus shengliensis* has been shown to be able to use a large variety of methoxylated aromatic compounds as substrates for methane generation termed methoxydotrophic methanogenesis (Mayumi et al. 2016). In contrast to acetogenic bacteria *M. shengliensis* is so far the only archaeon able to grow on methoxylated aromatic compounds. *M. shengliensis* ZC-1 was isolated from the Shengli oil field (China) and has been shown to use either methylated compounds like methanol (Cheng et al. 2007) or a large variety of methoxylated aromatic compounds (Mayumi et al. 2016) for growth. It was observed that during the growth of the organism the concentration of the methoxylated aromatic compound decreases while the concentration of the hydroxylated version of this compound increases, and CO_2_ and CH_4_ are formed. The exact metabolic pathway for growth on methoxy compounds is not known for methanogens so far. For acetogens, it is well-known that they use two methyltransferases, one corrinoid protein and one activating enzyme that recycles the corrinoid protein for transfer of the methyl group from methoxylated compounds (Kaufmann et al. 1997). Also, *M. shengliensis* features one gene cluster encoding two O-demethylases (Amam_00018/BP07_RS03255; Amam_00019/BP07_RS03250) similar to those of acetogens that might demethoxylate the methoxy compound and transfer the methyl group from the methoxy group to a cobalt-containing corrinoid protein (Fig. 5; Amam_00017/BP07_RS03260). The methyltransferase that shuttles the methyl group from the corrinoid protein into the methanogenesis pathway might be an MtrH-like methyltransferase (Amam_00021/BP07_RS03240), which is encoded in the same gene cluster as the O-demethylases, the corrinoid protein and the respective corrinoid activation protein (Amam_00022/BP07_RS03235). In contrast to growth on methanol, the methyl group is most likely transferred to tetrahydromethanopterin instead of coenzyme M by this methyltransferase (Kurth et al. unpublished results). Also, several transporters are encoded in that gene cluster that might be involved in the transport of the methoxylated aromatic compound into the cell and export of the hydroxylated aromatic compound.

### Methanogenesis from tertiary and quaternary amines

Choline (*N*,*N*,*N*-trimethylethanolamine) is a compound that is widely distributed in membrane lipids and has been shown to be used as a substrate for methanogenesis with ethanolamine as a product by five *Methanococcoides* strains (Watkins et al. 2012). Di- and monomethylethanolamine are metabolic intermediates in this pathway that temporarily accumulate. Both have also been shown to be a substrate for methanogenesis. Also, *Methanococcoides vulcani*, a marine methylotrophic methanogen isolated from a mud volcano, has been shown to use betaine, choline, and *N*,*N*-dimethylethanolamine for methanogenesis (L’Haridon et al. 2014). However, not all *Methanococcoides* strains can utilize choline (Watkins et al. 2012). Later on, it has also been shown that some marine *Methanococcoides* strains can use betaine (*N*,*N*,*N*-trimethylglycine) as a substrate for methanogenesis, partially demethylating it to *N*,*N*-dimethylglycine (Watkins et al. 2014). In contrast, *N*,*N*-dimethylglycine or sarcosine (*N*-methylglycine) could not be used as substrates in methanogenesis. Growth rates and yields during growth on betaine were similar to those with trimethylamine. However, betaine is only partially demethylated indicating that the yield per methyl group is significantly higher than with trimethylamine (Watkins et al. 2014). A tetramethylammonium-degrading methanogen (strain NaT1) was isolated from a sand sample obtained from Tokyo Bay (Tanaka 1994). Two further methanogen strains with the ability to utilize quaternary amines were isolated from an estuarine sediment (Ticak et al. 2015). Strain B1d is closely related to *Methanolobus vulcani* PL-12/MT and strain Q3c to *Methanococcoides* sp. PM1 and PM2. Strain Q3c was able to grow on tetramethylammonium and choline, while strain B1d was able to grow on betaine. B1d is the first quaternary amine-utilizing methanogen from the genus *Methanolobus* (Ticak et al. 2015). In conclusion, quaternary amines may serve as substrates for methanogenesis in marine environments.

The transport of quaternary amines is proposed to proceed via a betaine/choline/carnitine transporter (BCCT) or a homolog of the predicted trimethylamine permease (MttP) seen in other sequenced methylamine-utilizing methanogens (Ticak et al. 2015). Moreover, for quaternary amines like trimethylammonium, it is known that they are demethylated by a three-component enzyme system including a substrate-specific methyltransferase, a corrinoid-binding protein, and a CoM methyltransferase (Fig. 5; Asakawa et al. 1998; Ferguson et al. 2000). Next to the three-component methyltransferase systems for monomethylamine (MtmBCA), dimethylamine (MtbBCA) and trimethylamine (MttBCA) it has been described for *M. barkeri* that RamA, a 60-kDa monomeric ironsulfur protein, is required for ATP-dependent reductive activation of methylamine:CoM methyl transfer from all three methylamines (Ferguson et al. 2009). For the betaine consuming methanogen *Methanolobus vulcani* B1d it has been shown that the organism possesses the methyltransferase MtgB (FKV42_RS08545) that catalyzes betaine-dependent methylation of free cob(I)alamin (Creighbaum et al. 2019). Further, proteomic analysis revealed that MtgB, a corrinoid binding protein (FKV42_RS08550), a corrinoid reductive activation enzyme (FKV42_RS10455) and a methylcorrinoid:CoM methyltransferase (FKV42_RS10480) were highly abundant when *M. vulcani* B1d was grown on betaine relative to growth on trimethylamine. Energy conservation presumably follows what is known for methylamine or methanol dependent growth, using a membrane-bound respiratory chain involving Ech + Vho or Rnf and membrane-bound heterodisulfide reductase HdrDE.

## Conclusion

This review comprises an overview of unconventional substrates and pathways used by methanogenic archaea. Next to describing the involved organisms we mainly elaborated on the features of the different metabolic pathways focusing on the involved genes and enzymes. With the help of this overview and the compiled bioinformatic information, we provide helpful information for further research on different methanogenic pathways. For example, the key enzymes of the secondary alcohol metabolism of various methanogens, an iron depending alcohol dehydrogenase, has so far only been described bioinformatically but not biochemically. In addition, also the H_2_-dependent methylotrophic methanogenesis pathways of *Methanimicrococcus blatticola* and *Methanonatronarchaea* have not been studied so far. Bioinformatic analysis revealed that next to a membrane-bound heterodisulfide reductase, a methanophenazine-reducing hydrogenase in the case of *Methanimicrococcus blatticola* and a membrane-bound multisubunit hydrogenase in case of *Methanonatronarchaeum thermophilum* might be involved. However, this hypothesis still has to be proven. Moreover, the methyltransferase systems of methanogens using methylated sulfur compounds or tertiary and quaternary amines as substrates should be studied in more detail especially in view of substrate specificities of the involved enzymes. Another interesting research topic is the methanogenesis from methoxylated aromatic compounds. So far only the methanogen *Methermicoccus shengliensis* has been shown to make methane from those compounds. Nevertheless, the metabolic pathway of this organism has not yet been described in detail. In summary, we highlighted that there is potential for further research on the methanogenesis pathways mentioned in this review and for discovering further methanogenesis pathways on unconventional substrates.

## Data Availability

Not applicable
